# Anxiety, depression, and post-traumatic stress and associated risk factors among out-of-school girls in western Kenya

**DOI:** 10.1371/journal.pone.0323362

**Published:** 2025-05-14

**Authors:** Philip Spinhoven, Susan Nungo, Anna Maria van Eijk, Elizabeth Nyothach, Linda Mason, David Obor, Daniel Kwaro, Penelope A. Phillips-Howard, Garazi Zulaika

**Affiliations:** 1 Leiden University, Institute of Psychology, Leiden, The Netherlands; 2 Liverpool School of Tropical Medicine, Liverpool, United Kingdom; 3 Kenya Medical Research Institute, Kisumu, Kenya; 4 Safe Water and AIDS Project, Kisumu, Kenya; Columbia University Irving Medical Center, UNITED STATES OF AMERICA

## Abstract

**Background:**

Many adolescent girls drop out of school in sub-Saharan Africa. Mental health problems in this population and their risk factors are a neglected research area.

**Methods:**

This community-based cross-sectional survey studied 904 out-of-school girls

in rural western Kenya. Outcome variables were a positive screen for anxiety (GAD-7), depression (PHQ-A), post-traumatic stress disorder (PTSD; PCL-C), and a composite measure for overall symptom severity. Survey data were analysed with univariable and multivariable binary logistic and multiple linear regression analyses using SPSS 29.0.

**Findings:**

The prevalence of probable anxiety was 10.6%, of probable depression 15.9%, and of probable PTSD 18.0%. One of the three items on suicidal ideation or past suicide attempt was reported by 40.2% of girls. In multivariable analyses controlling for age, fear of sexual assault and functional limitations due to menstruation were uniquely associated with each of the outcome variables, and exposure to physical violence to each outcome except anxiety. Other risk factors showed a less consistent relationship with outcome. The cross-sectional study design precludes any temporal and causal inference for the reported significant associations.

**Conclusion:**

Out-of-school girls constitute a vulnerable group with high levels of PTSD and suicide risk. Multi-level and multi-sector interventions are needed to help these girls cope with their mental health problems and to address mutable risk factors such as gender-based partner and non-partner sexual and physical violence, poor menstrual hygiene, and poverty.

## Introduction

Mental disorders are a leading cause of disease-related burden worldwide. Around 50% of all mental disorders onset prior to age 18 [1]. As most mental disorders become chronic, this early onset can impede the transition into healthy adulthood. Mental disorders are associated with adverse outcomes such as high rates of school dropout, low economic productivity, and suicide, impacting individuals, families, and society at large [2]. Common mental disorders (CMDs) such as depression, anxiety, and post-traumatic stress disorder (PTSD) are prevalent among children and adolescents worldwide, with an estimated global prevalence of CMDs of 25–31% [3]. In sub-Saharan Africa (SSA) 23% of the population comprises adolescents between 10 and 19 years of age [4], and CMDs in this age group constitute a vast health problem [5]. Reliable data on the prevalence and risk factors associated with adolescent CMDs in SSA are limited.

A recent systematic review focusing solely on the prevalence of child and adolescent mental health problems in SSA reported that in the general population, median point prevalence rates were 26.9% (IQR: 20.1–31.1) for depression, 29.8% (IQR: 18.6–36.7) for anxiety disorders, 40.8% (IQR: 31.2–41.4) for emotional and behavioural problems, 21.5% (in only one study) for PTSD, and 20.8% (IQR: 13.2–23.6) for suicidal ideation [6]. Recently in Kenya, a nationally representative household survey [7] as part of the National Adolescent Mental Health Surveys (NAMHS) provided the first prevalence estimates for mental disorders in adolescents aged 10–17 based on a structured diagnostic instrument (the Diagnostic Interview for Children, Version 5) that applies criteria established in the Diagnostic and Statistical Manual of Mental Disorders (DSM-5) [8]. The prevalence of any mental disorder in the past four weeks was 9.4%: anxiety disorders were the most prevalent (3.4%), with lower prevalences of major depressive disorder (1.5%) and PTSD (0.9%). The prevalence of suicidal ideation in the past 12 months was 4.6% [7]. In the Global Burden of Disease Study 2019, mental disorders ranked as the second leading cause of disability among 10–24-year-olds in Kenya, accounting for 9.4% of the total health loss in this young population [9].

Most studies of mental health problems among Kenyan adolescents have been conducted in secondary schools, with those using self-reports reporting higher prevalence rates of CMDs than found by the NAMHS. In a stratified sample of all public secondary schools in Nairobi, clinical diagnostic scores for depression were observed in 43.7% and for anxiety in 12.9% of all students [10]. Other school-based Kenyan studies have found lower prevalences of depression ranging from 14.5% of pupils [11] to 22% [12]. One study in Nairobi found the prevalence of clinically significant depressive symptoms to be 26.4% and higher in girls than boys [13]; while another reported higher levels of depression symptoms (45.9% above clinical cut-off) and anxiety symptoms (38.0% above clinical cut-off) among older adolescents and girls [14]. It has been observed that during adolescence a significant sex disparity emerges in depression, with markedly higher prevalences in girls than in boys which is sustained until menopause [15,16]. Experts have hypothesized possible causes to be stemming from the onset of menstruation and the menstrual cycle, to biological differences and reproductive endocrinology, gendered societal norms, and higher rates of abuse and trauma [17].

Among urban adolescents, self-reported depression was associated with sexual and physical violence, adverse events, and less neighbourhood safety [12]. However, other studies suggest lower rates of anxiety and depression for adolescents living further away from urban Nairobi in more rural areas. Among adolescent girls and young women from multi-stage household random samples in an informal settlement and a peri-urban area in Kisumu County, the prevalence of anxiety/depression was 4% [18]. Examining emotional and behavioural problems in a stratified random sample of Kenyan adolescents in the country’s Central Province, 1.6% of the youths scored in the clinical range for anxious/depressed symptoms and 1.5% in the clinical range of withdrawn/depressed symptoms, with girls reporting higher scores than boys [19]. Higher levels of depression of 5.3% were found among secondary schoolgirls in rural western Kenya [20].

As far as we are aware, no studies have examined mental health problems in school-aged adolescent girls who dropped out of school in SSA. In this region, notably more girls (33.6%) than boys (28.9%) are out of school across primary and secondary education, with gendered disparities increasing as pupils get older [21]. As the transition from adolescence into adulthood is a critical period in the development of psychopathology, a substantial number of adolescents will develop a mental disorder during their school years. Studies in Western countries indicate that mental health problems increase the chance of dropping out of school [22] and that extended periods out of school increase the chance of permanent dropout because extended periods of missed schooling are correlated with higher rates of CMDs in students [23]. In other words, school dropout may be due to mental health problems, while mental health problems may be aggravated by dropping out of a school’s protective environment [24], further decreasing the chance of re-engagement.

In SSA countries, adolescent mental health morbidity is driven by a combination of sociodemographic factors (such as female gender, older age, impoverished living conditions, belonging to a minority tribe, being out-of-school, orphanhood, having a large number of siblings, being married or promised in marriage), medical/somatic factors (such as HIV/AIDS, substance/alcohol abuse, low physical activity) and adverse childhood experiences (such as exposure to violence, forced or unsafe sex, and maladaptive parental behaviour) [6,25]. Using the Global Burden of Disease comparative risk assessment framework for identifying risk factors for mental disorders among adolescents and young adults in Kenya, bullying and victimization, childhood sexual abuse, intimate partner violence and lead exposure were identified as important risk factors. In school-attending Kenyan adolescent girls from the same region as the present study, persistent quality of life problems were associated with adverse physical, sexual and emotional experiences and problems with coping with their monthly menstruation [20].

These findings underscore the importance of further exploring the role of mental health problems and associated risk factors in school dropout processes, as mental health problems may be more pronounced among those who drop out of school. As a first exploration of this topic in SSA, this study has the following two study aims: (a) to study the different types of mental health problems and their prevalence among out-of-school girls in western Kenya, and (b) to determine associated characteristics of different types of mental health problems. We hypothesized that adverse physical, sexual, and emotional experiences, as well as problems coping with menstrual hygiene would be associated with elevated levels of mental health problems among out-of-school girls.

## Methods

### Participants and study design

The present study is part of a cross-sectional study of factors associated with early school dropout and sexual and reproductive health among out-of-school girls in western Kenya. This analysis focuses on the prevalence of mental health problems and associated risk factors in the study population. The study is nested within the Kenya Medical Research Institute's (KEMRI) Health and Demographic Surveillance System (HDSS).

Eligibility criteria for girls included: having been out of school for more than three months, aged 10–19 years, reported to have reached menarche, living in the study area, and having no disability precluding participation. Identification of participants for recruitment was through KEMRI’s bi-annual HDSS household surveys. Girls were targeted for enrolment if listed in these surveys as “out of school” and under age 20. In addition, outreach was conducted through Community Health Promoters and other community informants to identify out-of-school adolescent girls who may have missed identification through the HDSS survey. Only one of the identified girls who was approached refused to participate. If girls had returned to school since the HDSS survey or had re-located out of the study site they were no longer eligible to participate.

### Study setting and data collection

The study covered rural regions in Siaya County, spreading across three sub-counties: Alego Usonga, Gem, and Rarieda in the western region of Kenya. A national adolescent and youth survey conducted in Siaya County found that around 4% of primary and 37% of secondary school students were out of school [26]. One recent randomised controlled trial in the area found that 11.3% of girls who entered secondary school dropped out prior to completion [27]. Data on demographics, schooling history, sexual history, interpersonal violence, menstruation, and work history were obtained through a sociodemographic survey questionnaire in the participants’ language of choice (English or Dholuo). Participant data were collected electronically on Android tablets, using a standardized case reporting form and Open Data Kit (ODK) software between November 2, 2021 and January 20, 2023. Participants self-administered the survey if literate; otherwise, a study counsellor led the survey. All personal identifying data were anonymized and number-coded at the source. All stored data were stripped of identifiers, and storage files were encrypted. A more detailed description of the study design and sampling procedures has been given elsewhere [28].

### Measures

The present study focused on CMDs in LMIC countries (i.e., depressive, anxiety, and post-traumatic stress symptoms). The Patient Health Questionnaire (PHQ-9) [29] modified for Adolescents (PHQ-A) [30], the 7-item Generalized Anxiety Disorder scale (GAD-7) [31] and the 17-item PTSD Checklist – Civilian Version (PCL-C) [32] were used to measure depressive, anxiety, and post-traumatic stress disorder symptoms, respectively. The PHQ-A also contains four additional questions on the number of days feeling depressed, functional impairments, suicidal ideation, and suicide attempts. Item scores are summated to derive a total score that ranges from 0 to 27 for PHQ-9, 0–21 for GAD-7, and 0–85 for PCL-C. For PHQ-9, total scores of 5–9, 10–14, and 15–27 indicate mild, moderate, and severe depressive symptoms, respectively. GAD-7 scores of 5–9, 10–14, and 15–21 indicate mild, moderate, and severe anxiety symptoms, respectively. The recommended optimal cut-off score of ≥ 10 for both PHQ-9 and GAD-7 [29,31], also applicable to SSA [33,34], was used to define positive screening for depression and anxiety. PHQ-9 and GAD-7 have been locally validated, showing good psychometric properties [35]. To screen for clinically elevated levels of PTSD symptoms, the total PCL-C score had to be equal to or greater than 44 [36]. Although the PCL-C has not been validated for use in LMIC countries, its successor based on DSM-5 criteria, the PCL-5, has been locally validated [37], and there is substantial agreement between both PCLs for probable PTSD based on DSM-IV and DSM-5 criteria [38]. In the present study, the internal consistency alphas for PHQ-9, GAD-7, and PCL-C were good to excellent: 0.83, 0.84, and 0.93, respectively.

In addition, a composite score for the severity of CMD symptoms was calculated based on a Principal Component Analysis (PCA) of standardized GAD-7, PHQ-9, and PCL-C total scores. Results showed that one component with an eigenvalue of 2.39 explained 79.5% of the variance, with component loadings varying from 0.87 to 0.92.

This exploratory study analysed mental health outcomes against a comprehensive set of putative risk factors. Risk factors explored included *individual characteristics* (age, marital status, not living with a biological parent, socio-economic status (SES)), schooling history (completed primary school, wanted to stop school, would like to return to school), sexual history (ever had sex, had forced sex, engaged in transactional sex, currently sexually active, currently pregnant, ever been pregnant), interpersonal violence (experienced intimate partner violence, being touched indecently, fear of sexual assault, exposure to physical violence, sexual harassment, emotional support), menstruation (early menarche, use of disposable pads, period duration, period severity, period related-cramps, functional limitations due to menstruation), and work history/other (engaged in paid chores or activities, used health care services). See S1 Table for a complete overview of variables, survey questions, answering formats, and coding for the statistical analyses.

### Statistical analyses

Survey data were analysed with logistic and linear regression analyses using SPSS 29.0. Outcome variables were a positive screen for anxiety, depression, and PTSD and the composite measure for severity of CMDs. For each outcome variable, risk variables with a p-value of < 0.05 in the univariable analyses were included in the multivariable backward regression models. These multivariable analyses were controlled for age given the developmental changes and challenges associated with the transition to late adolescence. In analysing positive screens using logistic regression, adjusted odds ratios (aORs) and their 95% confidence intervals (CIs) were generated. In analysing the severity of CMDs using linear regression, standardized beta, t-, and p-values were generated. The Hosmer-Lemeshow test (HL test) was used as a goodness of fit test for logistic regression and (adapted) R-squared as a goodness of fit test for linear regression. Assumptions underlying multiple linear regression (i.e., linearity, normality of residuals, homoscedasticity, and independence of error) were tested with scatterplots and the Shapiro-Wilk, Breusch-Pagan, and Durbin-Watson statistics, respectively. Before conducting the multivariable analyses, multicollinearity between variables was checked by calculating the variance inflation factors (VIF < 5).

We conducted several sensitivity analyses to validate the backward multivariable logistic and linear regression results. First, multiple imputation was used to produce five complete data sets from the incomplete data by modelling scale variables with a linear regression, and categorical variables with a logistic regression with the fully conditional specification model (FCS) of SPSS. Subsequently, each of the five completed data sets was analysed using logistic and linear regression, and results were combined to achieve inference. Secondly, we repeated the backward multivariable logistic and linear regression analyses including risk variables with a p-value of < 0.10 in the univariable analyses. Lastly, to examine whether violations of assumptions underlying the regression models threatened the integrity of our analyses, we repeated our multivariable logistic and linear regression analyses using a non-parametric approach using bootstrapping. More specifically, backward multivariable logistic and linear regression models, including all significant risk variables at p < 0.05 while controlling for age, were bootstrapped, and the results of the parametric and non-parametric analyses were compared. The sampling distribution of the estimator was estimated by resampling with replacement from the original entire sample using 1,000 bootstrap samples. These bootstrapped analyses were also executed, replacing the only continuous risk factor in our model (i.e., age) with two dummy-coded variables based on splitting the sample into three age groups of comparable size (<18 years, 18 years, > 18 years).

### Ethical considerations

The protocol and tools for this study were approved by KEMRI’s Scientific and Ethics Review Unit (SERU #4197) and the Liverpool School of Tropical Medicine’s (LSTM) Research and Ethics Committee (REC # 21–012). A research license was granted by the Kenyan National Commission for Science, Technology and Innovation (NACOSTI/P/21/12581). Participants were taken through the informed consent procedures if over age 18 or emancipated, before providing written consent. Written consent was obtained from parents and guardians of non-emancipated minors, with written informed assent of minors requested after parental consent was received. All field visits were conducted by female counsellors. Participants were given telephone numbers for the study safeguarding officer and referral locations were identified for girls who sought or required counselling. This manuscript adheres to the Strengthening the Reporting of Observational studies in Epidemiology guidelines (S2 Table). Additional information on considerations specific to inclusivity in global research is included in the supplement (S3 Checklist).

## Results

### Sample

Of the 934 girls identified, 915 completed the whole interview, of whom 904 also answered the GAD-7, PHQ-9, and PCL-C, constituting the present study sample. Of these, 368 (40.7%) were identified from the HDSS survey, and 536 (59.3%) were community referrals. Their median age was 18 (IQR: 17–19), and 46.2% came from the poorest SES (poorest two quintiles). As seen in Table 1, 73.7% were not living with a biological parent, and 33.8% were married/cohabitating or widowed. 65.5% completed primary school and started secondary school but did not complete it.

**Table 1:** Participant characteristics (n = 904).

CHARACTERISTICS	Total (N = 904)
Age (in years) median (IQR)	18.6 (17.5-19.4)
Married/cohabitating or widowed	306 (33.8%)
Not living with a biological parent	666 (73.7%)
Socio-economic status (poorest 2 vs less poor 3 quintiles)	418 (46.2%)
SCHOOLING HISTORY	
Completed primary school	592 (65.5%)
Wanted to stop school	103 (11.4%)
Would like to return to school	494 (54.6%)
SEXUAL HISTORY	
Ever had sex	768 (85.0%)
Forced or threatened to have sex	144 (15.9%)
Ever engaged in transactional sex	348 (38.5%)
Currently sexually active (past 6 months)	546 (60.4%)
Currently pregnant	157 (17.4%)
Ever been pregnant	698 (77.2%)
VIOLENCE	
Intimate Partner Violence (any)	140 (15.5%)
Indecently touched by a male (past 6 months)	150 (16.6%)
Felt scared would be sexually assaulted (past 6 months)	225 (24.9%)
Exposure to physical violence (past 6 months)	170 (18.8%)
Sexual harassment	136 (15.0%)
Emotional support of family or friends	737 (81.5%)
MENSTRUATION	
Early menarche (<13 years)	148 (16.4%)
Used disposable sanitary pads (last period)	796 (88.1%)
Period duration (≥7 days)	146 (16.2%)
Period severity heavy	251 (27.8%)
Had period-related pain or cramps	551 (61.0%)
Functional limitations due to menstruation	196 (21.7%)
WORK HISTORY / OTHER	
Done chores or activities for pay (past month)	146 (16.2%)
Used health care services (past 6 months)	582 (64.4%)
MENTAL HEALTH^a^	
Anxiety	96 (10.6%)
Depression	144 (15.9%)
Post-traumatic stress	163 (18.0%)
Suicide ideation: thoughts they would be better off dead or hurting themselves in some way (last 2 weeks)	267 (29.5%)
Suicide ideation: serious thoughts about ending their life (last month)	236 (26.1%)
Suicide attempt: tried to kill themselves (ever)	158 (17.5%)

Legend: ^a^ a positive screen for anxiety was derived from the Generalized Anxiety Disorder scale (score ≥10); for depression was derived from the Patient Health Questionnaire 9 item (PHQ-9) scale (score ≥10); and for post-traumatic stress was derived from the PCL-C PTSD Checklist - Civilian Version (score ≥44). Suicide ideation and attempts were collected from the PHQ-A.

### Prevalence of depression, anxiety and PTSD

In the total sample, PHQ-9 scores were mildly elevated (M = 4.99; SD = 4.89), and the prevalence of depression was 15.9% (95%CI: 13.6–18.3). GAD-7 scores fell within the normal range (M = 4.25; SD = 4.22), and the prevalence of anxiety was 10.6% (95%CI: 8.8–12.7). The level of PCL-C scores was moderate (M = 31.40; SD = 13.96), and the prevalence of PTSD was 18.0% (95%CI: 15.7–20.6). Of the girls, 669 (74.0%) had no positive screen, 110 (12.2%) had one positive screen, 82 (9.1%) had two positive screens, and 43 (4.8%) had three positive screens.

On the PHQ-A, 40.2% of the girls reported at least one of the three suicide items: 29.5% of the girls reported thoughts that they would be better off dead or of hurting themselves in some way on at least several days during the last two weeks; 26.1% reported having serious thoughts about ending their life during the previous month; and 17.5% reported that they had tried to kill themselves or made a suicide attempt at some point during their life (Table 1). Among the 26% of girls with at least one positive screen for PTSD, anxiety, or depression, the percentages of suicidal ideation and attempts were much higher and varied from 71.8% among positive screens for PTSD to 76.0% among those with anxiety and 79.2% among those with depression.

### Predictors of depression, anxiety and PTSD

The factors associated with the composite CMD factor scores derived from the PCA analysis, positive screen for depression on the PHQ-9, positive screen for anxiety on the GAD-7, and positive screen for PTSD on the PCL-C in the univariable analyses can be found in Tables 2–5. The severity of CMD symptoms and clinically elevated depression, anxiety, and PTSD symptom levels were all associated with a wide array of demographic, schooling history, sexual history, interpersonal violence, menstruation, and work history variables in the univariable analyses.

Before examining the predictive power of our linear regression model, possible violations of foundational assumptions were tested. The Durbin-Watson statistic of 2.026 suggested no autocorrelation, indicating independent errors; and predictor VIFs were well below the threshold of 5, indicating no multicollinearity concerns. However, the Shapiro-Wilk test did not substantiate the normality of the residuals (W (830) = 0.960, p < 0.001), and homoscedasticity was not confirmed via the Breusch-Pagan test (F (16,813) = 2.595, p < 0.001). In large sample sizes, both the Breusch-Pagan and Shapiro-Wilk tests are sensitive to small deviations from homoscedasticity and normality, respectively. Collectively, these tests indicated that complementary non-parametric analyses were needed to validate the results as not all assumptions for multiple linear regression were met.

The multivariable linear prediction model of severity of CMD symptoms was significant (F(9,848)=20.696, p < 0.001, R^2^ = 0.180, AR^2^ = 0.171). Moreover, the Hosmer and Lemeshow goodness of fit tests indicated a good fitting model with no difference between the observed and model-predicted values for clinically elevated anxiety (X^2^(8)= 11.167, p = 0.192, Nagelkerke R^2^ = 0.115), depression (X^2^(8)= 5.530, p = 0.700, Nagelkerke R^2^ = 0.127), and post-traumatic stress (X^2^(8)= 8.404, p = 0.395, Nagelkerke R^2^ = 0.133).

Higher levels of CMD symptoms were significantly associated with completion of only primary school, forced sex, current sexual activity, intimate partner violence, fear of sexual harassment, exposure to physical violence, and functional limitations due to menstruation. Clinically elevated depression, anxiety, and PTSD symptom levels were all associated with fear of sexual harassment and functional limitations due to menstruation (Tables 2–5). Depression and PTSD, but not anxiety, were related to exposure to physical violence. Higher age, poorer household SES, being forced or threatened into having sex, and experiencing menarche before age 13 were associated with elevated anxiety. Current sexual activity and period-related pain or cramps were associated with elevated depression. Intimate partner violence and sexual harassment were related to elevated post-traumatic stress.

**Table 2:** Standardized beta values and associated statistics as derived from univariable and backward multivariable linear regression models examining associated characteristics of a composite measure of anxiety, depression and post-traumatic stress as measured with the GAD-7, PHQ-9 and PCL-C.

	Univariable analyses			Multivariable analyses (incomplete data: n = 830)			Multivariable analyses (imputed data: n = 904)		
	β	t	p-value	aβ	t	p-value	aβ	t	p-value
Demographic characteristics									
Age (in years)	0.073	2.147	0.032	0.049	1.499	0.134	0.049	1.555	0.120
Married/cohabitating or widowed	0.059	1.775	0.076	(-)					
Not living with a biological parent	0.016	0.479	0.632	(-)					
SES (poorest 2 quintiles)	0.067	1.989	0.047	ns					
Schooling history									
Completed primary school	0.111	3.369	<0.001	0.078	2.408	0.016	0.079	2.541	0.011
Wanted to stop school	0.029	0.865	0.387	(-)					
Would like to return to school	0.072	2.137	0.033	ns					
Sexual history									
Ever had sex	0.112	3.382	<0.001	ns					
Forced or threatened to have sex	0.219	6.740	<0.001	0.100	2.868	0.004	0.093	2.855	0.004
Ever engaged in transactional sex	0.088	2.665	0.008	ns					
Currently sexually active (<6 months)	0.148	4.482	<0.001	0.069	2.009	0.045	0.082	2.494	0.013
Currently pregnant	0.065	1.957	0.051	(-)					
Ever been pregnant	0.073	2.202	0.028	ns					
Interpersonal violence									
Intimate partner violence	0.255	7.909	<0.001	0.099	2.665	0.008	0.090	2.555	0.011
Indecently touched by male (<6 months)	0.107	3.242	0.001	ns					
Fear of sexual assault (<6 months)	0.236	7.280	<0.001	0.129	3.764	<0.001	0.130	4.021	<0.001
Exposure to physical violence (<6 months)	0.273	8.536	<0.001	0.148	4.295	<0.001	0.162	5.000	<0.001
Sexual harassment	0.210	6.436	<0.001	0.069	1.976	0.049	0.079	2.380	0.017
Emotional support of family or friends	0.015	0.436	0.663	(-)					
Menstruation									
Early menarche (<13yrs)	0.057	1.711	0.087	(-)					
Used disposable sanitary pads	-0.021	-0.645	0.519	(-)					
Period duration (≥7 days)	0.087	2.621	0.009	ns					
Period severity - heavy	0.065	1.963	0.050	(-)					
Period-related pain or cramps	0.104	3.147	0.002	ns					
Functional limitations due to menstruation	0.190	5.797	<0.001	0.129	3.917	<0.001	0.114	3.669	<0.001
Work history / other									
Chores or activities for payment or something in return (<1 month)	0.081	2.426	0.158	(-)					
Healthcare service use (<6 months)	0.031	0.946	0.344	(-)					

Legend: Statistically significant at p < 0.05 in bold. Abbreviations: β = beta; aβ = adjusted beta; GAD-7 = Generalized Anxiety Disorder scale; PHQ-9 = Patient Health Questionnaire; PCL-C = PTSD Checklist - Civilian Version; SES = socio-economic status. (-) = variable ≥ 0.05 in univariable analyses and not included in the multivariable prediction model; ns = variable no longer significant after controlling for all other variables in the multivariable prediction model. Assumptions: Durbin-Watson statistic = 2.026; Shapiro-Wilk test: (W (830)=0.960, p < 0.001); Breusch-Pagan test: (F (16,813)=2.595, p < 0.001). CMD multivariable model fit statistics: (F (9,848) =20.696, p < 0.001, R^2^ = 0.180, AR^2^ = 0.171).

**Table 3:** Odds ratios and adjusted odds ratios as derived from univariable and backward multivariable logistic regression models examining associated characteristics of a positive screen for anxiety as measured with the GAD-7^a^.

	Anxiety Case	Non-Anxiety Case	Univariable analyses			Multivariable analyses (incomplete data: n = 830)			Multivariable analyses (imputed data: n = 904)		
	N (%)	N (%)	OR	95%CI	p-value	aOR	95%CI	p-value	aOR	95%CI	p-value
Demographic characteristics											
Age (in years) median (IQR)	18.0 (18-19)	18.0 (17-19)	1.37	(1.26-1.67)	0.002	1.37	(1.10-1.71)	0.005	1.29	(1.06-1.58)	0.011
Married/cohabitating or widowed	34 (35.4)	272 (33.7)	1.08	(0.69-1.68)	0.731	(-)					
Not living with a biological parent	69 (71.9)	597 (73.9)	0.90	(0.56-1.45)	0.672	(-)					
SES (poorest 2 quintiles)	55 (59.8)	363 (46.3)	1.72	(1.11-2.68)	0.015	1.84	(1.13-2.98)	0.014	1.82	(1.14-2.90)	0.013
Schooling history											
Completed primary school	59 (61.5)	533 (66.0)	1.21	(0.79-1.88)	0.380	(-)					
Wanted to stop school	11 (11.7)	92 (11.6)	1.01	(0.52-1.96)	0.980	(-)					
Would like to return to school	56 (59.6)	438 (55.3)	1.19	(0.77-1.84)	0.431	(-)					
Sexual history											
Ever had sex	88 (91.7)	680 (84.2)	2.07	(0.98-4.37)	0.057	(-)					
Forced or threatened to have sex	33 (34.4)	111 (13.7)	3.29	(2.06-5.24)	<0.001	1.85	(1.04-3.31)	0.037	2.26	(1.35-3.77)	0.002
Ever engaged in transactional sex	45 (46.9)	303 (37.5)	1.47	(0.96-2.25)	0.076	(-)					
Currently sexually active (<6 months)	71 (74.0)	475 (58.8)	1.99	(1.24-3.21)	0.005	ns					
Currently pregnant	18 (18.8)	139 (17.2)	1.11	(0.64-1.91)	0.705	(-)					
Ever been pregnant	76 (79.2)	622 (77.0)	1.14	(0.68-1.91)	0.629	(-)					
Interpersonal violence											
Intimate partner violence	27 (28.1)	113 (14.0)	2.41	(1.48-3.92)	<0.001	ns					
Indecently touched by male (<6 months)	22 (22.9)	128 (15.8)	1.58	(0.95-2.64)	0.080	(-)					
Fear of sexual assault (<6 months)	45 (46.9)	180 (22.3)	3.08	(1.99-4.75)	<0.001	2.11	(1.27-3.53)	0.004	2.42	(1.52-3.86)	<0.001
Exposure to physical violence (<6 months)	36 (37.5)	134 (16.6)	3.02	(1.92-4.75)	<0.001	ns					
Sexual harassment	28 (29.2)	108 (13.4)	2.67	(1.64-4.33)	<0.001	ns					
Emotional support of family or friends	81 (84.4)	656 (81.2)	1.25	(0.70-2.23)	0.448	(-)					
Menstruation											
Early menarche (<13 years)	24 (25.0)	124 (15.3)	1.84	(1.11-3.03)	0.017	1.89	(1.06-3.38)	0.032	1.96	(1.14-3.37)	0.014
Used disposable sanitary pads	82 (85.4)	714 (88.4)	0.77	(0.42-1.41)	0.401	(-)					
Period duration (≥7 days)	16 (16.7)	130 (16.1)	1.04	(0.59-1.84)	0.884	(-)					
Period severity - heavy	28 (29.2)	223 (27.6)	1.08	(0.68-1.72)	0.746	(-)					
Period-related pain or cramps	73 (76.0)	478 (59.2)	2.19	(1.34-3.57)	0.002	ns					
Functional limitations due to menstruation	41 (42.7)	155 (19.2)	3.14	(2.02-4.88)	<0.001	2.14	(1.26-3.64)	0.005	2.68	(1.68-4.30)	<0.001
Work history / other											
Chores or activities for payment or something in return (<1 months)	17 (17.7)	129 (16.1)	1.13	(0.65-1.98)	0.661	(-)					
Healthcare service use (<6 months)	71 (74.0)	511 (63.2)	1.65	(1.02-2.66)	0.040	ns					

Legend: Statistically significant at p < 0.05 in bold. Abbreviations: OR = odds ratio; aOR = adjusted odds ratios; GAD-7 = Generalized Anxiety Disorder scale; ^a^ case = a score of 10 or higher on the GAD-7; SES = socio-economic status. (-) = variable ≥ 0.05 in univariable analyses and not included in the multivariable prediction model; ns = variable no longer significant after controlling for all other variables in the multivariable prediction model. Model fit statistics: Hosmer and Lemeshow goodness of fit = (X^2^(8)= 11.167, p = 0.192, Nagelkerke R^2^ = 0.115).

**Table 4:** Odds ratios and adjusted odds ratios as derived from univariable and backward multivariable logistic regression models examining associated characteristics of a positive screen for depression as measured with the PHQ-9^a^.

	Depression Case	Non-Depression Case	Univariable analyses			Multivariable analyses (incomplete data: n = 858)			Multivariable analyses (imputed data: n = 904)		
	N (%)	N (%)	OR	95%CI	p-value	aOR	95%CI	p-value	aOR	95%CI	p-value
Demographic characteristics											
Age (in years) median (IQR)	18.0 (17-19)	18.0 (17-19)	1.09	(0.94-1.26)	0.252	1.01	(0.87-1.19)	0.858	1.01	(0.87-1.18)	0.901
Married/cohabitating or widowed	59 (41.0)	247 (32.5)	1.44	(1.00-2.08)	0.050	(-)					
Not living with a biological parent	113 (78.5)	553 (72.8)	1.36	(0.89-2.09)	0.155	(-)					
SES (poorest 2 quintiles)	68 (49.3)	350 (47.4)	1.08	(0.75-1.55)	0.690	(-)					
Schooling history											
Completed primary school	84 (58.3)	508 (66.8)	1.44	(1.00-2.07)	0.050	(-)					
Wanted to stop school	15 (10.7)	88 (11.8)	0.90	(0.50-1.60)	0.714	(-)					
Would like to return to school	86 (61.4)	408 (54.7)	1.32	(0.91-1.91)	0.142	(-)					
Sexual history											
Ever had sex	134 (93.1)	634 (83.4)	2.66	(1.36-5.21)	0.004	ns					
Forced or threatened to have sex	40 (27.8)	104 (13.7)	2.43	(1.59-3.69)	<0.001	ns					
Ever engaged in transactional sex	72 (50.0)	276 (36.3)	1.75	(1.22-2.51)	0.002	ns					
Currently sexually active (<6 months)	109 (75.7)	437 (57.5)	2.30	(1.53-3.46)	<0.001	2.11	(1.35-3.28)	<0.001	2.28	(1.48-3.51)	<0.001
Currently pregnant	32 (22.2)	125 (16.4)	1.45	(0.94-2.25)	0.095	ns					
Ever been pregnant	119 (82.6)	579 (76.2)	1.49	(0.94-2.36)	0.092	ns					
Interpersonal violence											
Intimate partner violence	42 (29.2)	98 (12.9)	2.78	(1.83-4.22)	<0.001	ns					
Indecently touched by male (<6 months)	35 (24.3)	115 (15.1)	1.80	(1.17-2.77)	0.007	ns					
Fear of sexual assault (<6 months)	61 (42.4)	164 (21.6)	2.67	(1.84-3.88)	<0.001	1.93	(1.26-2.97)	0.003	2.33	(1.57-3.47)	<0.001
Exposure to physical violence in the last 6 months	48 (33.3)	122 (16.1)	2.61	(1.76-3.89)	<0.001	1.66	(1.06-2.62)	0.043	1.80	(1.17-2.77)	0.007
Sexual harassment	40 (27.8)	96 (12.6)	2.66	(1.74-4.06)	<0.001	1.64	(0.99-2.78)	0.054	1.84	(1.15-2.93)	0.011
Emotional support of family or friends	121 (84.0)	616 (81.1)	1.23	(0.76-1.99)	0.400	(-)					
Menstruation											
Early menarche (<13 years)	22 (15.3)	126 (16.6)	0.91	(0.55-1.48)	0.699	(-)					
Used disposable sanitary pads	124 (86.1)	672 (88.4)	0.81	(0.48-1.37)	0.434	(-)					
Period duration (≥7 days)	31 (21.5)	115 (15.1)	1.54	(0.99-2.40)	0.057	ns					
Period severity - heavy	51 (35.4)	200 (26.3)	1.53	(1.05-2.24)	0.026	ns					
Period-related pain or cramps	106 (73.6)	445 (58.6)	1.97	(1.33-2.94)	<0.001	1.60	(1.02-2.52)	0.043	1.61	(1.04-2.50)	0.032
Functional limitations due to menstruation	52 (36.1)	144 (18.9)	2.42	(1.64-3.55)	<0.001	1.60	(1.02-2.51)	0.040	1.64	(1.06-2.52)	0.026
Work history / other											
Chores or activities for payment or something in return (<1 month)	32 (22.2)	114 (15.0)	1.62	(1.04-2.51)	0.032	ns					
Healthcare service use (<6 months)	97 (67.4)	485 (63.8)	1.17	(0.80-1.71)	0.416	(-)					

Legend: Statistically significant at p < 0.05 in bold. Abbreviations: OR = odds ratio; aOR = adjusted odds ratios; PHQ-9 = Patient Health Questionnaire; ^a^ case = a score of 10 or higher on the PHQ-9; SES = socio-economic status. (-) = variable ≥ 0.05 in univariable analyses and not included in the multivariable prediction model; ns = variable no longer significant after controlling for all other variables in the multivariable prediction model. Model fit statistics: Hosmer and Lemeshow goodness of fit = (X^2^(8)= 5.530, p = 0.700, Nagelkerke R^2^ = 0.127).

**Table 5:** Odds ratios and adjusted odds ratios as derived from univariable and backward multivariable logistic regression models examining associated characteristics of a positive screen for post-traumatic stress as measured with the PCL-C^a^ (n = 904).

	PTSD Case	Non-PTSD Case	Univariable analyses			Multivariable analyses (incomplete data: n = 842)			Multivariable analyses (imputed data: n = 904)		
	N (%)	N (%)	OR	95%CI	P-value	aOR	95%CI	p-value	aOR	95%CI	p-value
Demographic characteristics											
Age (in years) median (IQR)	18.0 (17-19)	18.0 (17-19)	1.17	(0.97-1.28)	0.119	1.08	(0.93-1.25)	0.333	1.07	(0.93-1.24)	0.330
Married/cohabitating or widowed	63 (38.7)	243 (32.8)	1.29	(0.91-1.83)	0.153	(-)					
Not living with a biological parent	118 (72.4)	548 (74.0)	0.92	(0.63-1.35)	0.682	(-)					
SES (poorest 2 quintiles)	78 (48.8)	340 (47.5)	1.05	(0.75-1.48)	0.772	(-)					
Schooling history											
Completed primary school	96 (58.9)	496 (66.9)	1.41	(0.99-2.00)	0.051	(-)					
Wanted to stop school	19 (11.9)	84 (11.6)	1.03	(0.61-1.75)	0.913	(-)					
Would like to return to school	102 (63.7)	392 (54.0)	1.50	(1.05-2.13)	0.025	ns					
Sexual history											
Ever had sex	149 (91.4)	619 (83.5)	2.10	(1.17-3.75)	0.012	ns					
Forced or threatened to have sex	38 (23.3)	106 (14.3)	1.82	(1.20-2.76)	0.005	ns					
Ever engaged in transactional sex	78 (47.9)	270 (36.4)	1.60	(1.14-2.25)	0.007	ns					
Currently sexually active (<6 months)	115 (70.6)	431 (58.2)	1.72	(1.19-2.49)	0.004	ns					
Currently pregnant	31 (19.0)	126 (17.0)	1.15	(0.74-1.77)	0.539	(-)					
Ever been pregnant	130 (79.8)	568 (76.7)	1.20	(0.79-1.82)	0.393	(-)					
Interpersonal violence											
Intimate partner violence	48 (29.4)	92 (12.4)	2.94	(1.97-4.40)	<0.001	1.84	(1.15-2.95)	0.012	1.73	(1.10-2.70)	0.016
Indecently touched by male (<6 months)	35 (21.5)	115 (15.5)	1.49	(0.97-2.27)	0.066	(-)					
Fear of sexual assault (<6 months)	70 (42.9)	155 (20.9)	2.85	(1.99-4.07)	<0.001	1.79	(1.18-2.69)	0.006	1.96	(1.33-2.89)	<0.001
Exposure to physical violence (<6 months)	59 (36.2)	111 (15.0)	3.22	(2.21-4.70)	<0.001	1.96	(1.26-3.04)	0.003	2.04	(1.34-3.10)	<0.001
Sexual harassment	46 (28.2)	90 (12.1)	2.84	(1.89-4.27)	<0.001	1.74	(1.08-2.81)	0.023	1.74	(1.11-2.48)	0.012
Emotional support of family or friends	137 (84.0)	600 (81.0)	1.24	(0.78-1.96)	0.360	(-)					
Menstruation											
Early menarche (<13 years)	28 (17.2)	120 (16.2)	1.07	(0.68-1.69)	0.759	(-)					
Used disposable sanitary pads	140 (85.9)	656 (88.5)	0.79	(0.48-1.29)	0.348	(-)					
Period duration (≥7 days)	36 (22.1)	110 (14.8)	1.63	(1.07-2.48)	0.024	ns					
Period severity - heavy	49 (30.1)	202 (27.3)	1.15	(0.79-1.66)	0.470	(-)					
Period-related pain or cramps	110 (67.5)	441 (59.5)	1.41	(0.99-2.02)	0.060	(-)					
Functional limitations due to menstruation	55 (33.7)	141 (19.0)	2.17	(1.49-3.15)	<0.001	1.68	(1.14-2.55)	0.013	1.67	(1.12-2.48)	0.012
Work history / other											
Chores or activities for payment or something in return (<1 months)	38 (213.3)	108 (14.6)	1.78	(1.17-2.70	0.007	ns					
Healthcare service use (<6 months)	116 (71.2)	466 (62.9)	1.46	(1.01-2.11)	0.047	ns					

Legend: Statistically significant at p < 0.05 in bold. Abbreviations: OR = odds ratio; aOR = adjusted odds ratios; PCL-C = PTSD Checklist - Civilian Version; ^a^ = a score of 44 or higher on the PCL-C; SES = socio-economic status. (-) = variable ≥ 0.05 in univariable analyses and not included in the multivariable prediction model; ns = variable no longer significant after controlling for all other variables in the multivariable prediction model. Model fit statistics: Hosmer and Lemeshow goodness of fit = (X^2^(8)= 8.404, p = 0.395, Nagelkerke R^2^ = 0.133).

### Sensitivity analyses

Results of multivariable logistic and regression analyses of the imputed complete data sets yielded comparable results as analyses of the dataset with missing data. All prediction models were highly significant and identified the same significant risk factors (Tables 2–5). The only difference was that the borderline significant association of sexual harassment with elevated depression in the incomplete data set (p = 0.054) became significant in the complete data set (p = 0.011) (Table 4). Moreover, backward multiple logistic and linear regression models with the inclusion of significant risk variables at p < 0.10 yielded identical prediction models as models including only risk factors significant at p < 0.05. Finally, the bootstrapped results of backward multivariable logistic and linear regression models, including all significant risk variables at p < 0.05 while controlling for age, showed the same significant risk factors for the severity of CMD symptoms and elevated levels of anxiety, depression, and post-traumatic stress as identified by the parametric analyses (see Table 6). The same results emerged when substituting age as a numeric variable with ‘age 18’ and ‘age >18’ contrasted with ‘age <18’. More specifically, the significant positive association of age with elevated anxiety levels re-appeared as significantly higher levels of anxiety in girls older than 18 years compared to girls younger than 18 years (18 years of age: OR = 1.87 (CI = 0.098–3.58); 19 years or older: OR = 2.13 (CI = 1.149–3.948)). Together, these results suggest that the violation of assumptions underlying linear regression did not critically affect the integrity of the linear prediction model of CMD severity.

**Table 6:** Unstandardized beta values and adjusted odds ratios as derived from bootstrapped backward multivariable linear and logistic regression models examining associated characteristics of a composite measure of anxiety, depression and post-traumatic stress and a positive screen on the GAD-7, PHQ-9 and PCL-C.

	Composite measure			GAD-7			PHQ-9			PCL-C		
	β	95%CI	p-value	aOR	95%CI	p-value	aOR	95%CI	p-value	aOR	95%CI	p-value
Demographic characteristics												
Age	0.040	-0.008-0.086	0.096	1.37	1.14-1.69	0.002	1.02	0.86-1.21	0.843	1.10	0.96-1.30	0.201
Married/cohabitating or widowed	(-)						(-)			(-)		
SES (poorest 2 quintiles)	ns			1.86	1.18-3.18	0.011	(-)			(-)		
Schooling history												
Completed primary school	0.145	0.002-0.274	0.036	(-)			(-)			(-)		
Would like to return to school	(-)			(-)			(-)			ns		
Sexual history												
Ever had sex	ns			(-)			ns			ns		
Forced or threatened to have sex	0.299	0.071-0.527	0.008	2.52	1.24-4.10	0.009	ns			ns		
Ever engaged in transactional sex	ns			ns			ns			ns		
Currently sexually active (<6 months)	0.167	0.037-0.299	0.013	ns			2.16	1.42-3.64	0.002	ns		
Ever been pregnant	ns			ns			(-)			(-)		
Interpersonal violence												
Intimate partner violence	0.252	0.022-0.489	0.032	ns			ns			1.78	1.09-2.87	0.025
Indecently touched by male (<6 months)	ns			ns			ns			(-)		
Fear of sexual assault (<6 months)	0.290	0.123-0.449	0.002	2.30	1.42-3.92	<0.001	2.04	1.31-3.17	0.002	1.83	1.17-2.78	0.003
Exposure to physical violence (<6 months)	0.391	0.205-0.574	<0.001	ns			1.71	1.04-2.69	0.029	1.98	1.24-3.07	0.002
Sexual harassment	0.229	-0.040-0.425	0.028	ns			ns			1.86	1.09-3.05	0.009
Menstruation												
Early menarche(<13yrs)	(-)			1.91	0.99-3.30	0.026	(-)			(-)		
Period duration (≥7 days)	ns			ns			(-)			ns		
Period severity - heavy	(-)						ns			(-)		
Period-related pain or cramps	ns			ns			1.63	1.05-2.61	0.030	(-)		
Functional limitations due to menstruation	0.279	0.107-0.444	<0.001	2.61	1.59-4.52	<0.001	1.67	1.02-2.59	0.024	1.66	1.05-2.53	0.015
Work history / other												
Chores or activities for payment or something in return (<1 months)	(-)			(-)			ns			ns		
Healthcare service use (<6 months)	(-)			ns			(-)			ns		

Legend: Statistically significant at p < 0.05 in bold. Abbreviations: β = beta; aOR = adjusted odds ratios; GAD-7 = Generalized Anxiety Disorder scale; PHQ-9 = Patient Health Questionnaire; PCL-C = PTSD Checklist - Civilian Version; (-) = variable ≥ 0.05 in univariable analyses and not included in the multivariable prediction model; ns = variable no longer significant after controlling for all other variables included in the multivariable prediction model

## Discussion

The first aim of the present study was to examine mental health problems and their prevalence among out-of-school girls in rural western Kenya. Below, we will discuss the prevalence of these disorders and symptoms in more detail.

### Suicidal ideation and suicide attempts

In our sample of Kenyan adolescent girls, 29.5% reported thoughts that they would be better off dead or hurting themselves in some way on at least several days during the last two weeks, 26.1% reported having serious thoughts about ending their life during the previous month, and 17.5% reported that they had tried to kill themselves or made a suicide attempt at some point during their life. 40.2% of the girls reported at least one of these three suicide items, and about 72%-79% of the girls did so with a positive screen for anxiety, depression, or PTSD. These figures are higher than the 20.8% for suicidal ideation reported in the systematic review of the prevalence of child and adolescent mental health problems in SSA [6]. They are also higher than in the few extant studies on suicide risk in Kenya reporting a suicide risk of 20.0% among adolescent students joining secondary schools in Nairobi county [11] and an overall presence of different types of suicidal ideation of 22.6% among Kenyan high school, college and university students [39]. The much lower prevalence rates of 4.6% for suicidal ideation, 2.4% for suicide planning, and 1.0% for suicide attempts found in the nationally representative household survey of NAHMS [7] may be due to questions on suicidal behaviour being interviewer-administered in this study. The reporting of sensitive behaviour tends to be lower in interviewer-administered surveys compared to self-administered surveys, thus stigma could have resulted in a lower reporting of these behaviours.

Many adolescents with suicidal ideation will not make a suicide attempt as fear of death is a powerful barrier to action. In addition to strong suicidal ideation, personal factors as well as practical factors are necessary to overcome this barrier [40]. The HDSS revealed only one female suicide among 526 deaths in adolescents and young females in the catchment area of our study [41], although it is possible that suicide, as the cause of mortality, is not reliably registered. In Kenya, attempted suicide is still a misdemeanour and, upon conviction, is punishable by imprisonment or a fine [42]. Worldwide, suicide is the second leading cause of death in the 15–19 years age group [43]. As suicidal ideation and past suicide attempts are so prevalent among out-of-school girls and, in particular, in out-of-school girls with CMD, recognition and destigmatization of this problem are urgently needed. It is noteworthy that during data collection no adverse events occurred in administering the suicide items of the PHQ-A, and many girls even welcomed the possibility of sharing these negative personal experiences with the study counsellor.

### Post-traumatic stress disorder

The lifetime prevalence of PTSD worldwide is estimated to be 3.9%, rising to 5.6% among the trauma-exposed [44]. Prevalence rates range from 0.5% to 14.5% across countries, which may stem from regional variations in trauma exposure types [44]. In a recent meta-analysis specifically focusing on SSA, the pooled prevalence of current symptoms consistent with a probable diagnosis of PTSD was 25% [45]. Omitting studies with persons younger than 18 did not change prevalence estimates. In a meta-analysis of mental health problems in children and adolescents in SSA, a comparable estimate of 21.5% based on only one study was reported [6]. Higher prevalence rates for PTSD in SSA are to be expected as PTSD is paradigmatic for a disorder intrinsically linked with trauma exposure, and persons living in SSA are disproportionally affected by individual and population-level trauma exposure (e.g., armed conflicts, natural disasters, traffic accidents, and interpersonal violence) [45].

When looking specifically at Kenya, the recent NAHMS survey found the adolescent PTSD prevalence rates to be 0.9% in the past 4 weeks and 1.3% in the past 12 months [7]. However, studies using self-administered surveys among Kenyan adolescents have found higher rates of disorder that are more aligned with those reported across SSA. One comparative study reported rates for both full symptoms of PTSD and partial symptoms of PTSD in Kenyan adolescent in Nairobi at 12% and 8%, respectively [46]. In a second study among a representative sample of secondary school students in Nairobi, symptoms of partial and full PTSD were notably higher at 34.8% and 50.5%, respectively [47]. We found that 18% of our out-of-school girls had a positive screen for PTSD, which aligns more closely with studies using self-administered questionnaires [46,47] and evidence of PTSD symptoms across SSA [44,45], and is in contrast to what was found in the interviewer-moderated NAHMS household survey [7]. We note that it is possible that the even higher rates of PTSD found in adolescents living in Nairobi may not be generalizable to other Kenyan adolescents, as rural adolescents may be less exposed to accidents or violent crimes than those living in urban centres.

### Anxiety and depression

The prevalence rates for anxiety (10.6%) and depression (15.9%) in our study are lower than other self-reported rates reported for children and adolescents in SSA (26.9% and 29.8%, respectively) [6] and for students in secondary schools in Nairobi, varying from 12.9% [10] to 37.7% [14] for anxiety and from 14.5% [11] to 45.9% [14] for depression. To note, the studies of Osborn and colleagues [14] and Mokaya and colleagues [11] also used the PHQ and the Osborn study used the GAD-7. However, as previously noted, the interviewer-based Kenyan NAMHS among adolescents reported much lower prevalence rates (5.6% (past 12 months) and 3.4% (past 4 weeks) for anxiety and 2.0% (past 12 months) and 1.5% (past 4 weeks) for major depressive disorder) [7]. Nonetheless, the most direct comparisons of the present study results are with a sample of adolescent girls from secondary schools recruited in the same area of western rural Kenya also using the PHQ-9 with the same cut-off [20] and a sample of adolescent girls and young women from an informal settlement and a peri-urban area in Kisumu County (geographically adjacent to the current study’s site) [18]. These comparisons show a much higher prevalence of depression in out-of-school girls (15.9% in this current study versus 5.3% [20] and 4.0% [18], respectively) in line with our hypothesis that the prevalence of mental health problems in this group will be higher in comparison to a normative school-going sample of adolescent girls.

A recent meta-analysis addressed the impact of urbanicity on the prevalence of depression in the general adult and adolescent demographics of developing countries [48]. Results showed that the relationship between urbanicity and depression within developing countries changed over time. Older studies showed a greater prevalence of depression in rural populations, while newer studies reported a greater prevalence of depression in urban populations. This same transition seen in developed countries may be occurring in LMIC, wherein the risk factors of urbanization that have contributed to the greater urban depression are becoming prevalent as many people in these countries move to urban centres. Further research is needed to investigate whether urbanicity is related to the prevalence of CMDs among adults and adolescents in Kenya.

### Over- and under-reporting of mental health problems

The dearth of studies on mental health and numerous measurement tools complicate efforts to assess to what extent our estimated prevalence rates are affected by assessment type. Prevalence rates for mental disorders in Kenya based on a semi-structured interview using DSM-5 criteria in a nationally representative household survey in which 97.6% of the adolescents were currently attending school [7] were substantially lower than estimated rates based on self-report questionnaires administered to secondary school-going and adolescent out-of-school populations. As validated mental health screening questionnaires and designated cut-off thresholds do not give a clinical diagnosis, only a probable diagnosis of depression, anxiety, or PTSD can be derived. Among studies with a semi-structured interview reference standard, pooled PHQ-9 sensitivity and specificity at the standard cut-off value of ≥ 10, were 0.85 (0.79 to 0.89) and 0.85 (0.82 to 0.87), respectively [49]. In studies that compared the PCL with structured diagnostic interviews, sensitivity decreased from 0.85 to 0.39 and specificity increased from 0.73 to 0.97 for cutoffs ranging from 30 to 60 [50]. In short, how these screening tools perform is critically affected by the given population’s prevalence of the disorder and the selected cut-off values. Conversely, the applicability of DSM-5 diagnostic criteria to persons living in other cultures has been questioned, given culturally relevant symptoms or signs may be missed or underreported in the DSM-5 [51]. Additionally, due to the fluid character of psychopathology in adolescents and young adults, symptom measures assessing differences in severity may be more appropriate than psychiatric diagnoses representing fixed categorisations.

The reporting of sensitive experiences and behaviours tend to be lower in interviews than in self-reports because of stigma, a culture of silence surrounding physical and sexual violence, or fear of potential social, moral, or even legal consequences [52]. The strikingly diverging rates of suicidal ideation and suicide attempts discussed above could be a consequence of these phenomenon. There is a dearth of studies on the cultural-sensitive applicability of questionnaires and interviews for mental health problems for adolescents and young adults in LIMC and their concordance [53]. Collecting these types of data with robust monitoring and referral systems has been defined by the WHO as a priority for improving global adolescent health [54].

The second aim of our study was to determine to what extent demographic, schooling history, sexual history, interpersonal violence, menstruation, and work history variables are associated with different types of mental health problems.

### Gender-based violence

In particular, the association of the severity and presence of CMDs with multiple interpersonal adverse events (i.e., forced sex, intimate partner violence, physical abuse, fear of sexual assault, harassment for sex) in the present study stands out. These results are in line with those in high-income countries, showing that adverse childhood and adolescent experiences contribute to mortality and morbidity and have long-lasting effects on mental health [55]. They also concur with some recent studies of adolescent girls in Kenya reporting that the experience of sexual violence with both intimate partners and non-intimate partners is associated with elevated levels of anxiety and depression [18], and that the experience of sexual and physical violence and adverse life events is associated with elevated levels of depression [12].

In a recent meta-analysis on the prevalence of child sexual abuse worldwide, it was found that the pooled prevalence estimate for females below age 18 years of age was 9% for forced intercourse, 13% for contact abuse, and 31% for non-contact abuse [56]. No significant differences between studies concerning prevalent estimates of child sexual abuse regarding regional distribution and degree of development of the country were observed. Our results on sexual abuse in out-of-school girls show higher prevalence rates of forced sex (15.9%) and comparable rates of contact abuse (16.6%) and non-contact abuse (fear of sexual assault and sexual harassment) (27.8%). The rates for these types of sexual abuse were quite comparable to the rates previously found in in-school girls from the same region (14.9%, 14.6%, and 41.2%, respectively) [20]. Among girls with probable anxiety, depression, or PTSD the rate of forced sex varied between 23.3 and 34.4%.

As PTSD prevalence is associated with type of traumatic exposure and interpersonal traumatic events (such as physical and sexual abuse and intimate partner violence [57]), adolescent out-of-school girls exposed to frequent gender-based violence are especially vulnerable to developing PTSD.

### Menstruation

Aside from gender-based violence, mental health problems were also consistently associated with functional impairments due to menstruation, while early menarche showed a unique association with anxiety and period-related pain or cramps with depression. These associations all point to a relationship between inadequate menstrual health and hygiene and detrimental mental health outcomes, as also observed in secondary school girls in the same region [20]. To date, little focus has been given to how menstrual cycle-related symptoms and endocrinology are potentially associated with increased affective disorders in adolescent girls, with experts calling for more research on this topic and on the longer-term developmental sequelae into adulthood [17]. Additionally, a lack of products for menstrual hygiene management, awareness, and facilities, as well as stigma, are pervasive problems preventing good management of menstruation in LMIC, and the lack of money to buy sanitary products likely aggravate these problems [58]. Our study adds to this limited evidence, indicating further research on menstruation and mental health is warranted.

### Study strengths and limitations

The strengths of the present study include a focus on an understudied population of out-of-school girls, a representative sample for a rural area, an adequate sample size, and the use of internationally recognised and standardized self-report scales for CMDs with locally validated reliability. However, our results should be interpreted in light of several limitations in addition to the challenges with mental health assessments aforementioned. First, the cross-sectional study design precludes any temporal and causal inference for the reported significant associations. Cross-sectional designs preclude causal inferences [52], and confounding variables may distort or mask the effect of another third variable on the mental health outcomes investigated (e.g., other negative life events prevalent among adolescents in Kenya such as bullying [53] or neighbourhood safety [19]). Second, our findings for a rural area may not be generalisable to adolescent out-of-school girls in more urban settings. Third, comparing study results on mental health is hampered by differences in methodology, sample characteristics, type of screening tools used, translation and validation of tools, how and by whom assessments were administered, and other unexplained variations. Fourth, different types of trauma exposure were not systematically assessed, and emotional abuse with its significant health effects remained underexposed.

### Implications of the study findings for clinical care and health policy

A recent meta-analysis of interventions explicitly aiming to improve menstrual, sexual, reproductive, and mental health among out-of-school girls identified only seven intervention studies. Five interventions constituted educational programs, two were cash interventions, and strikingly, none were aimed at improving mental health status, illustrating an important gap in public health services for this vulnerable young population [59].

However, when looking at mental health problems for general adolescent populations, a recent review of mental health interventions for adolescents in SSA showed several diverse interventions applicable across various resource-constrained settings [60]. For a review of the effectiveness of specific interventions for anxiety, depression, and PTSD, see [61,62], and for a review of interventions to prevent or reduce gender-based violence in women and girls, see [63,64]. While the available number of studies are limited, they show positive effects of psychological and social interventions on adolescent anxiety, depression, and PTSD and for preventing gender-based violence and managing gender-based violence-related symptoms in these populations.

As far as we are aware, no research on the prevention or treatment of suicidal ideation and suicide attempts in SSA is available. Suicide remains a traditionally taboo subject in SSA, and psychoeducation is critical in combatting the stigma associated with suicide [42]. As suicide is associated with population-level and individual-level clinical and psychosocial risk factors, multi-level prevention and intervention strategies focussing on population-, community-, and individual-level targets are necessary while considering the local and cultural relevance [65].

It may be advisable to adopt a multi-level and multi-sectorial approach with interventions simultaneously addressing individual, community, and population-level factors to address mental health disorders and the multiplicity of risk factors girls face. Gender-responsive schools with adequate water, sanitation, and hygiene (WASH) facilities, and family level and community-based sensitization and interventions are needed to mitigate the impact of these factors. For female adolescents, mental health literacy, prevention, and health promotion could be combined with sexual and reproductive health programs [9,66]. Such an approach may result in a broader range and more enduring positive outcomes than a single intervention (e.g., see [67,68]). One major challenge to improving access to such psychological and psychosocial interventions is the dearth and geographic maldistribution of skilled mental health professionals in SSA [69], with any available service professionals predominantly concentrated in urban settings. Moreover, girls’ ability to access these providers is heavily influenced by their age, as legal structures often require family involvement and girls’ lack of disposable income hinders their ability travel to or pay for these services independently. Consequently, local health care and support systems for adolescent girls in rural western Kenya is nearly non-existent.

Task sharing to the front line (i.e., delegating care tasks to community or primary care-based non-specialist workers) has been advocated to address this problem [70]. In the treatment of depression, task-shared psychological interventions produced a more considerable reduction in depression severity and a higher rate of response and remission than control conditions [71]. Especially for reaching out-of-school girls, task-sharing could be a promising approach as the engagement of trusted and trained lay persons may be acceptable and feasible, and could help to break isolation, destigmatize mental health problems, and lower barriers to accessing the psychological and psychosocial help this vulnerable group of girls requires.

## Conclusion

Out-of-school girls constitute a vulnerable population, as evidenced by their elevated levels of anxiety, depression, PTSD and suicidal ideation. Multi-level and multi-sector approaches are warranted for mental health destigmatization, recognition and support; for developing and delivering age-appropriate service provision for mental health problems; and for addressing prevalent risk factors such as gender-based violence, partner and non-partner sexual and physical violence, poor menstrual hygiene, school attrition and poverty.

## Supporting information

S1 Table Overview of survey questions, response categories and coded values for statistical analyses. (DOCX)

S2 Table STROBE Checklist. (DOCX)

S3 Checklist Inclusivity in Global Health Research. (DOCX)
