# Supplementary material for: Anxiety, depression, and post-traumatic stress and associated risk factors among out-of-school girls in western Kenya
Source: PLoS One. 2025 May 14;20(5):e0323362. doi: 10.1371/journal.pone.0323362 (PMC12077770; doi:10.1371/journal.pone.0323362)
Supplement: S1 Table — (DOCX) [file pone.0323362.s001.docx]

**Supplementary Information:**

| **S1 Table: Overview of survey questions, response categories and coded values for statistical analyses** | | | |
| --- | --- | --- | --- |
| **Variables** | **Survey question** | **Response categories** | **Response values** |
| **Demographic characteristics** | | | |
| Age (in years) median (IQR) | Interviewer collected date of birth | Day, Month, Year | Calculated age from date of visit and date of birth. |
| Married/cohabitating or widowed | What is your marital status? (Cohabiting means that you are living with your partner but with no formal marriage certificate) | Single (not cohabiting) / Married /  Cohabiting / Widowed / Other | 1=Married/Cohabiting/Widowed; 0= Other |
| Not living with a biological parent | Who are the people you live with now? | Birth mother / Grandmother / Other female adults / Birth father / Grandfather / Other male adults / Younger brothers / Older brothers / Younger sisters / Older sisters/ Own child / Husband/Partner:  (Yes/No per response option) | 1= Birth mother or birth father; 0=Other |
| Socio-economic status (poorest 2 quintiles vs 3 less poor quintiles) | Composite of absolute index of household indicators pulled from the Kenya Medical Research Institute’s (KEMRI) Health and Demographic Surveillance System (HDSS) bi-annual household census. |  | 1 = poorest two wealth quintiles  0 = richer three wealth quintiles |
| **Schooling history** | | | |
| Completed primary school | Have you ever been to school?  What level of schooling did you reach by the time you stopped? | Started primary did not complete / Finished Primary / Started secondary did not complete | 1=Never been to school/started but did not complete primary school; 0=Other |
| Did want to stop school | Did you want to stop school? | Yes/No | 1=Yes; 0=No |
| Would like to return to school | Did you want to go back to school after pregnancy?  Did you want to go back to school after you got married?  Did you want to go back to school after you left? | Yes/No | 1. At least once yes; 0=Never yes |
| **Sexual history** | | | |
| Ever had sex | Have you ever had sex with a man or boy? | Never/ One time/ A few times/ Many times | 1= One time/ A few times/ Many times; 0=Never |
| Forced or threatened to have sex | Has a man or boy ever forced or threatened you to make you have sex? | Never/ One time/ A few times/ Many times | 1= One time/ A few times/ Many times; 0=Never |
| Ever engaged in transactional sex | Does the boy or man give you something for having sex with him? | Yes/No | 1=Yes; 0=No |
|  | If yes, what? | Money/ Food/drink / House items /School items/ Help with exams/ Pads for your monthly period/ Less beatings/bad things / Clothes/things/ Other:  (Yes/No per response option) | 1=Yes; 0=No |
| Currently sexually active | In the last 6 months, have you had sex, or been forced or tricked to have sex with a man or a boy? | Yes/No | 1=At least once yes; 0=Never yes |
|  | Do you currently have sex with a boy or man who you consider to be your boyfriend, partner, lover or husband? | Yes/No | 1=Yes; 0=No |
| Currently pregnant | Are you currently pregnant? | Yes/No | 1=Yes; 0=No |
| Ever been pregnant | Have you ever been pregnant? | Yes/No | 1=Yes; 0=No |
| **Interpersonal violence** | | | |
| Intimate partner violence  (only among those with a sexual partner in the past 6 months) | In the last 6 months did the man or boy physically hurt you in any of the following ways ?(check all that apply): | Push you, shake you, or throw something at you/ Slap you/ Twist your arm or pull your hair/ Punch you with his fist or with something that could hurt you/ Kick you or drag you or beat you/ Try to choke you or burn you on purpose/ Threaten or attack you with a knife, gun, or any other weapon: (Yes/No per response option) | 1=At least once yes; 0=Never yes |
|  | In the last 6 months has the man or boy sexually hurt you in any of the following ways ?(check all that apply): | Ever forced you to have sexual intercourse against your will/ Ever forced you to perform any other sexual acts against your will: (Yes/No per response option) | 1=At least once yes; 0=Never yes |
|  | In the last 6 months has the man or boy emotionally hurt you in  any of the following ways ?(check all that apply): | Say or do something to humiliate you in front of others/ Threaten to hurt or harm you or someone close/ Insult you or make you feel bad about yourself: (Yes/No per response option) | 1=At least once yes; 0=Never yes |
| Touched indecently by a boy/man in the last 6 months | In the last 6 months, have you been touched indecently by a boy/man? | Never/ Just once/ A few times/ Many times | 1= Just once/ A few times/ Many times; 0=Never |
| Fear of sexual assault in the last 6 months | In the last 6 months, have you ever felt scared you would be sexually assaulted? | Never/ Just once/ A few times/ Many times | 1= Just once/ A few times/ Many times; 0=Never |
| Exposure to physical violence in the last 6 months | In the last 6 months, has anyone hit, slapped, kicked or hurt you physically? | Never/ Just once/ A few times/ Many times | 1= Just once/ A few times/ Many times; 0=Never |
| Sexual harassment | Do boys or men harass you for sex? | Never/ A few times/ Many times | 1= A few times/ Many times/ 0=Never |
| Emotional support of family or friends | Do you talk to or visit your parents or trusted family for help and support? | Never/ Sometimes/ Regularly | 1. At least once sometimes/ regularly  0= Never on both questions |
|  | Do you talk to or visit your trusted friends for help and support? | Never/ Sometimes/ Regularly | 1. At least once/sometimes/ regularly  0= Never on both questions |
| **Menstruation** | | | |
| Early menarche <13yrs | How old were you when you first started your monthly period? | User entered integer | 1= less then 13 years; 0=Other |
| Used disposable sanitary pads | In the past 6 months have you ever used sanitary pads? | Yes/No | 1=Yes; 0=No |
| Period duration 7+ days | How many days did you bleed during your most recent period? | User entered integer | 1= more than or equal to 7 days; 0=Other |
| Period severity heavy | Was it heavy, normal or light? | Heavy/ Normal/ Light | 1=Heavy; 0=Normal/light |
| Period-related pain or cramps | Did you have pains or cramps? | Yes/No | 1=Yes; 0=No |
| Functional limitations due to menstruation | Did your period stop you doing things? | Yes/No | 1=Yes; 0=No |
|  | If yes, what did you stop doing? | House work/ Employment work/ Shamba/farm work/ Other work/ Other | 1=Yes; 0=No per response option |
| **Work history / other** | | | |
| Chores or activities for payment or something in return in the last month | In the last month, have you done any chores or activities for which you got paid or were given something in return? | Yes/No | 1=Yes; 0=No |
| Health care service use in past 6 months | In the past 6 months did you go or need to use health care services? | Yes/No | 1=Yes; 0=No |
